# Understanding Health Care Workers' Anxieties in a Diversifying World

**DOI:** 10.1371/journal.pmed.0040319

**Published:** 2007-11-13

**Authors:** Karen Daniels, Leslie Swartz

## Abstract

The authors discuss a new qualitative study on the challenges that physicians face in providing culturally appropriate care.

We live in an age of evidence-based medicine [1], and with good reason. It is important to be able to evaluate the likely impact of various interventions, and it is no longer acceptable to practice health care on the basis of received wisdom and anecdotal evidence, which has convincingly been shown to be subject to serious bias [2].

One of the dangers of the evidence revolution in health care, however, is that the emphasis on an actuarial approach (see Glossary) to evaluating health interventions may obscure the influence of more complex interactions between patients and health professionals [3]. The impact of these interactions upon health care outcomes may be idiosyncratic and rather difficult to measure. Two important ways in which evidence-based medicine has attempted to account for these interactions are (1) the increasing emphasis on what is variously called effectiveness (as opposed to efficacy) research, and (2) the project to turn evidence into clinical practice guidelines so that patients receive consistent care.

What is clear, however, is that neither of these developments can ever account fully for the nuances in style and attitude that may differentiate one clinician from another, or one group of clinicians from another. We know, for example, that users of services may prefer to be treated by clinicians of a particular gender or background [4,5], and that they may at different times and in different contexts wish to be treated by someone very similar to or very different from oneself [6].

## A New Qualitative Study

Qualitative research is the best way to access the nuances of difference that may occur across different contexts and individuals. A new qualitative study of how health professionals respond to ethnic diversity among their patients, by Joe Kai and colleagues, provides an excellent example of the contribution that qualitative research can make to our understanding of the messier, more complex side of health care [7].

**Linked Research Article**

This Perspective discusses the following new study published in *PLoS Medicine*:

Kai J, Beavan J, Faull C, Dodson L, Gill P, et al. (2007) Professional uncertainty and disempowerment responding to ethnic diversity in health care: a qualitative study. PLoS Med 4(11): e323. doi:10.1371/journal.pmed.0040323

From a qualitative study, Joe Kai and colleagues have identified opportunities to empower health professionals to respond more effectively to challenges in their work with patients from diverse ethnic communities.

The study involved 18 focus groups that included 106 health professionals from a variety of disciplines working with an ethnically diverse patient population in the Midlands region of the United Kingdom. Using the constant comparison method to analyze the interview transcripts, a number of themes emerged. Professionals experienced discomfort and uncertainty in responding to the needs of patients whose ethnicity was different to that of the professional, and they worried about the possibility of showing culturally inappropriate behaviour.

## Implications of the Study

Kai and colleagues' study provides a useful way to think about the range of anxieties felt by health workers in the face of the global phenomenon of increasing diversity in populations, and hence in health care users and providers. The authors call on us to consider the implications of the fact that despite the existence of many (probably numbering in the hundreds) manuals and guidelines on health care practice in the context of cultural diversity, the “real-world” experiences of health workers may be marked by uncertainty, confusion, disempowerment, and even fear.

Many manuals on intercultural health care implicitly position health care workers as ethnocentric (i.e., looking at the world through the lens of their own culture) and uninformed about diversity, therefore in need of training. But Kai and colleagues' study suggests that even when health care workers are well aware of intercultural issues, they may feel trapped by their sense of inability to do the right cultural thing. Perhaps it is time for those of us interested in cultural issues in health to consider far more carefully the complexity of personal and political positions in which heath care workers, such as the participants in this study, find themselves to be trapped. The problem for at least some health care workers is no longer that they know nothing about cultural diversity, but rather that they feel disempowered, as these authors show, by what they *do* know. This finding suggests, in fact, the opening of a new research agenda. We need to know far more about how health workers in a range of contexts experience and do their work, not from a position of naïveté about cultural issues but as consumers of a wealth of literature exhorting them to be more culturally sensitive.

## Health Care in a Diversifying World

Kai and his colleagues' study appears at a particular moment in world history. Not only is there increasing mobility of populations across the globe at an unprecedented rate (including large migrations of health workers [8]), there is also a sense in popular media in a post-9/11 world that diversity may be dangerous [9]. The “other,” once cosily romanticized in many texts about culture and health, is now sometimes portrayed by both governments and media as potentially dangerous, not only to individuals but also to the fabric of entire societies. Within health care, furthermore, empowerment of patients, and the erosion of the automatic authority once associated with medical doctors in particular, have led to a situation in which health workers can no longer rely on an easy sense of their own authority.

**Glossary**

**Actuarial approach:** An approach to health care that calculates interventions in terms of their benefit, risk, and cost. Within this approach, best practice is that which can be said to balance effectiveness and efficiency.

**Effectiveness:** The extent to which an intervention produces a beneficial result under the usual “real world” conditions

**Efficacy:** The extent to which an intervention produces a beneficial result under ideal conditions (e.g., in a trial)

**Constant comparison method:** An interactive approach to data sampling, collection, and analysis in qualitative research. As new data are collected they are compared to current data. Assumptions, queries, and gaps in the current data set are clarified through further data collection.

Global shifts, therefore, combine with issues more local to health care in creating an environment that is much more uncertain for both health care professionals and their clients than it once was, and this uncertainty mirrors and reproduces much of what is felt in society as a whole. Readers of this journal who are concerned with providing the best possible health care in a changing world would do well to consider carefully the implications of the article by Kai and his colleagues, and to provide more examples of fine-grained research that should help us move to a more sophisticated understanding of what culturally sensitive health care can and should be.
